# CAN Algorithm: An Individual Level Approach to Identify Consequence and Norm Sensitivities and Overall Action/Inaction Preferences in Moral Decision-Making

**DOI:** 10.3389/fpsyg.2020.547916

**Published:** 2021-01-13

**Authors:** Chuanjun Liu, Jiangqun Liao

**Affiliations:** ^1^Department of Sociology and Psychology, School of Public Administration, Sichuan University, Chengdu, China; ^2^Department of Psychology, School of Social Sciences, Tsinghua University, Beijing, China

**Keywords:** CAN algorithm, moral dilemma, moral decision-making, CNI model, multinomial process tree model, process dissociation

## Abstract

Recently, a multinomial process tree model was developed to measure an agent’s consequence sensitivity, norm sensitivity, and generalized inaction/action preferences when making moral decisions (CNI model). However, the CNI model presupposed that an agent considers *consequences*—*norms*—generalized *inaction*/*action* preferences sequentially, which is untenable based on recent evidence. Besides, the CNI model generates parameters at the group level based on binary categorical data. Hence, the *C*/*N*/*I* parameters cannot be used for correlation analyses or other conventional research designs. To solve these limitations, we developed the CAN algorithm to compute *norm* and *consequence* sensitivities and overall *action*/*inaction* preferences algebraically in a parallel manner. We re-analyzed the raw data of the original CNI model to test the methodological predictions. Our results demonstrate that: (1) the *C* parameter is approximately equal between the CNI model and CAN algorithm; (2) the *N* parameter under the CNI model approximately equals *N*/(1 − *C*) under the CAN algorithm; (3) the *I* parameter and *A* parameter are reversed around 0.5 – the larger the *I* parameter, the more the generalized inaction *versus* action preference and the larger the *A* parameter, the more overall action *versus* inaction preference; (4) tests of differences in parameters between groups with the CNI model and CAN algorithm led to almost the same statistical conclusion; (5) parameters from the CAN algorithm can be used for correlational analyses and multiple comparisons, and this is an advantage over the parameters from the CNI model. The theoretical and methodological implications of our study were also discussed.

## Introduction

Traditional moral dilemmas pit utilitarianism against deontology. Take the well-known problem of the trolley car as an example. An uncontrollable trolley car is rushing toward five workers who do not notice this emergency. There is a sidetrack and only one worker there, not noticing this emergency either. The only way to save the five workers is to pull the switch and let the trolley car run into the sidetrack. If that occurs, the one worker on the sidetrack will die and the five workers on the main track will be saved. The principle of utilitarianism is followed if the agent chooses to pull the switch because it achieves greater benefits than costs (Mill, 1872; Bentham, 1996). The principle of deontology is followed if the agent chooses not to pull the switch because harming the innocent is not allowed according to moral norms (Rawls, 1971; Kant and Gregor, 1997).

However, interpretations of the paradigms of traditional moral dilemmas are ambiguous (Gawronski and Beer, 2017; Gawronski et al., 2017). In the trolley-car dilemma, there could be three reasons why the agent is likely to pull the switch. The first is that the agent has weaker norm sensitivity, and is less averse to the sacrificing utilitarian proposal. The second is that the agent has a stronger consequence sensitivity, and finds the result of pulling the switch to be considerably beneficial. The third is that the agent wants to pull the switch and have a stronger generalized action (or weaker generalized inaction) preference irrespective of the norms and consequences behind it. The paradigm of the traditional dilemma cannot dissociate these three possibilities. Thus, we cannot tell whether norm sensitivity, consequence sensitivity, or generalized action/inaction preference matter in the agent’s moral decision-making.

To solve this ambiguity, Gawronski et al. (2017) developed a multinomial processing tree (MPT) model to dissociate the three possible interpretations stated above. First, they expanded the conceptual manipulations of utilitarianism and deontology. They addressed the manipulation limitation of a traditional dilemma. “Utilitarian” presupposes that the observed behavior is sensitive to consequences, which requires experimental manipulations of consequences. “Deontological” presupposes that the observed behavior is sensitive to moral norms, which requires experimental manipulations of moral norms.

Hence, four types of dilemmas involving different combinations of consequences and norms must be considered (Gawronski and Beer, 2017; Gawronski et al., 2017). That is, dilemmas in which a: (a) a *proscriptive* norm opposes the proposed behavior, and the benefits of behavior for overall wellbeing are *greater* than the costs of behavior; (b) a *proscriptive* norm opposes the proposed behavior, and the benefits of behavior for overall wellbeing are *smaller* than the costs of behavior; (c) a *prescriptive* norm endorses the proposed behavior, and the benefits of behavior for overall wellbeing are *greater* than the costs of behavior; (d) a *prescriptive* norm endorses the proposed behavior, and the benefits of behavior for overall wellbeing are *smaller* than the costs of behavior. In the case of the traditional moral dilemma, only one combined situation (proscriptive norm and benefits greater than costs) was included and not the other three combined situations (proscriptive norm and benefits smaller than costs; prescriptive norm and benefits greater than costs; prescriptive norm and benefits smaller than costs).

Second, they used an MPT to depict the mental processes of the agent’s moral judgment. The multinomial processing tree predicting action *versus* inaction responses in moral dilemmas with proscriptive and prescriptive norms and consequences involving benefits of action that are greater or smaller than the costs of action. The consequences sensitivity, norm sensitivity and generalized inaction versus action preferences are hypothesized to be sequentially processed, which corresponds to different response patterns in moral decision-making (for details, please refer to the Figure 1 of Gawronski et al., 2017).

Together with the MPT model, the model equations are attached. The sum of probabilities of action and inaction in each dilemma is 1, so we have listed only the equations for action probability. To simplify the equations, let *p*(action| proscriptive norm, benefits > costs) be *p*1, let *p*(action| proscriptive norm, benefits < costs) be *p*2, let *p*(action| prescriptive norm, benefits > costs) be *p*3, let *p*(action| prescriptive norm, benefits < costs) be *p*4, and same hereinafter.

(1) p1=C+(1-C)×(1-N)×(1-I)

(2) p2=(1-C)×(1-N)×(1-I)

(3) p3=C+(1-C)×N+(1-C)×(1-N)×(1-I)

(4) p4=(1-C)×N+(1-C)×(1-N)×(1-I)

With this model, three parameters could be dissociated using maximum likelihood statistics: consequence sensitivity (*C*), norm sensitivity (*N*), and generalized inaction *versus* action irrespective of consequences and norms (*I*). Hence, the model was termed the “CNI model.” Gawronski et al. (2017) provided protocols with a MultiTree program (Moshagen, 2010) to generate *C*/*N*/*I* parameters^1^.

## Methodological Limitations of the CNI Model

The CNI model contributes to the literature because it claimed to dissociate the three possibilities if the agent makes decisions in a traditional moral dilemma. Therefore, the CNI model can be used to solve several inconsistent findings, such as whether incidental emotions affect moral judgment and how (Gawronski et al., 2018). However, recently Baron and Goodwin demonstrated several theoretical problems underlying the CNI model, such as the prohibition of deontological rules (for details, see Baron and Goodwin, 2020). In the present study, we want to highlight some methodological limitations of the CNI model and to solve them using a new algorithm.

First, the CNI model is not suitable for correlation and regression analyses. This limitation has been stressed by Gawronski et al. (2017) themselves. *C*/*N*/*I* parameters are at the group level rather than at the individual level. They think individual level estimates are unreliable because the number of 24 moral dilemma trials are too small. The small number of observations often leads to poor model fit at the individual level. Thus, the CNI model cannot be used in studies aiming to discuss correlations.

Second, the CNI model can only compare the differences between two parameters and one parameter to a specific value. It is inapplicable if multiple comparisons beyond two conditions need to be made. The *C/N/I* parameters are generated with the multiTree software (Moshagen, 2010). Means of goodness-of-fit statistics are used to evaluate the adequacy of the model in describing the data. The model fit can reflect whether the empirically observed probabilities are significantly statistically deviated from the probabilities predicted by the model. When testing differences in two parameters across groups, these parameters are forced to be equal. As a result, if the model fit is significantly deviated, the tested two parameters are statistically different. On the contrary, if the model fit is not significantly deviated, the tested two parameters are statistically equal. It is similar when comparing the parameter to a specific value. When testing differences between a parameter to a specific value, that parameter is forced to be equal to that value. As a result, if the model fit is significantly deviated, the tested parameter is statistically different to that value. On the contrary, if the model fit is not significantly deviated, the tested parameter is statistically not different to that value. Thus, comparisons beyond two conditions are inapplicable in the CNI model. More recently, we notice that they have overcome the above limitations and provided an individual level model (Korner et al., 2020). However, they still donot fix the following fatal defect of their model.

**FIGURE 1:**
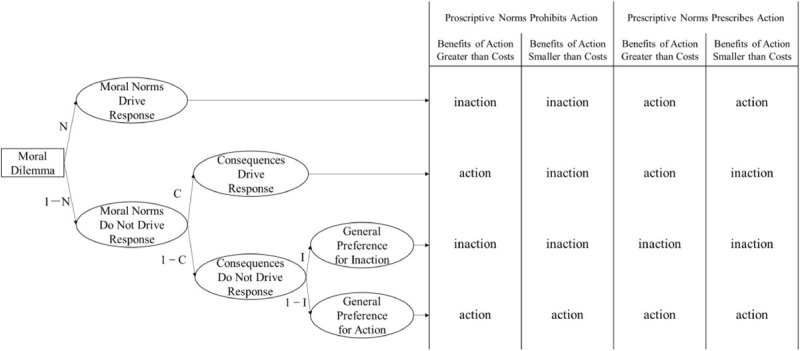
NCI model. The positions of the *C* parameter and *N* parameter are exchanged based on the original CNI model.

Lastly (but most importantly), the CNI model hypothesizes that the agent first considers whether the consequences of the proposed behavior are beneficial, then, considers whether the proposed behavior is allowed by moral norms, and finally, considers strategies of either generalized action or inaction irrespective of consequences or norms. This *priori* hypothesis is untenable for two reasons. First, if the agent sequentially considers the decision principles, s/he would not feel dilemmatic when *norms* prohibit action while *consequences* advocate action. The agent will feel dilemmatic only if s/he is simultaneously considering *norm* and *consequence* principles in conflicted situations. Thus, the agent is more likely to simultaneously (rather than sequentially) activate his/her *norm* and *consequence* principles. Recently, Bago and De Neys (2019) also speculated that people may simultaneously have deontological and utilitarian intuitions and that behavioral decisions are driven by the strengths of these two intuitions. In other words, people are intuitively sensitive to moral norms and consequences simultaneously but not sequentially. Second, there could be other sequential processing patterns even if the agent is in a sequential mindset. The sequential processing patterns could be *N* → *C* → *I* (first considering *norms*, if not, then considering *consequences* and finally, considering strategies of generalized action/inaction), *I* → *C* → *N* (first obtaining a generalized action/inaction preference, then revising it by the *consequences* principle and, finally, revising it by the *norms* principle), *I* → *N* → *C* (first obtaining a generalized action/inaction preference, then revising it by the *norms* principle and, finally, revising it by the *consequences* principle), and other potential sequential response patterns. Taking the *N* → *C* → *I* pattern as an example, named the “NCI model,” see Figure 1.

With the NCI model, we can use model equations to depict the response probabilities of the four combined dilemma situations, too:

(5) p1=(1-N)×C+(1-N)×(1-C)×(1-I)

(6) p2=(1-N)×(1-C)×(1-I)

(7) p3=N+(1-N)×C+(1-N)×(1-C)×(1-I)

(8) p4=N+(1-N)×(1-C)×(1-I)

Gawronski et al. (2017) discussed this in their footnote 7: all the reported effects were replicated with the NCI model, and the only differences were that some marginally significant effects in the CNI model became statistically significant with the NCI model. Therefore, they did not discuss further the differences between the NCI model and CNI model. However, if the CNI model and NCI model depicted the observed data equally, then equations (1) to (4) and equations (5) to (8) would be statistically identical to generate the parameters. Taking the *N* parameter as an example, it can be transformed from equations (1) to (4) so that *N* = (−*p*1 − *p*2 + *p*3 + *p*4)/(2 − *p*1 + *p*2 − *p*3 + *p*4), and also be transformed from equations (5) to (8) into *N* = (−*p*1 − *p*2 + *p*3 + *p*4)/2. If the CNI model and NCI model are statistically equivalent, these two *N* parameters should be equal. After conversion, it turns out that *p*2 − *p*1 = *p*3 − *p*4. In the same way, to transform the *C* parameter based on the equations for the CNI model and NCI model, it turns out that *p*1 + *p*2 = *p*3 + *p*4. Combining these two transformed equations, it would turn out that *p*2 = *p*3 and *p*1 = *p*4. These conversions imply that the CNI model and NCI model would generate the same *N* and *C* parameters only if *p*2 = *p*3, and *p*1 = *p*4. However, this precondition obviously has a very low empirical possibility.

The first two limitations of CNI model were due to the fact that the parameters were recorded at a group level rather than at an individual level. The last but the most fatal limitation of the CNI model was due to the fact that the CNI model presupposed the agent was sequentially rather than parallelly considering the *norm* and *consequence* principles. Given these methodological limitations, we tried to develop a new algorithm to identify the agent’s *norm* and *consequence* sensitivities and overall action/inaction preferences.

## CAN Algorithm

The traditional moral dilemma is varied into four parallel versions by manipulating the potential moral principles of norms and consequences, and the action is prohibited or advocated by *norm* and *consequence* principles. Thus, we can use a common algebraically subtracting strategy to generate *C* and *N* parameters. This strategy is commonly used in the literature, such as loyalty/nepotism could be computed as the amount of participants who rewarded their friend minus the amount they punished their friend (Talhelm et al., 2014). With respect to the *A* parameter, we used an aggregate mean strategy to measure the overall action *versus* inaction preferences, which is explained below.

With respect to the *C* parameter, if individuals are sensitive to consequences, they are more likely to approve a proposal under the conditions of benefits greater than costs than under the conditions of benefits smaller than costs. Therefore, the sensitivity of consequences under proscriptive norm conditions could be represented by *p*1 − *p*2, and the sensitivity of consequences under prescriptive norm conditions could be represented by *p*3 − *p*4. Hence, the c*onsequences* sensitivity is represented by the mean value of the two conditions, i.e., *C* = (*p*1 − *p*2 + *p*3 − *p*4)/2.

With regard to the *N* parameter, the sensitivity of norms under conditions of benefits greater than costs could be represented by *p*3 − *p*1; the sensitivity of norms under conditions of benefits smaller than costs could be represented by *p*4 − *p*2. Thus, the *norms* sensitivity is represented by the mean value of the two conditions, i.e., *N* = (*p*3 − *p*1 + *p*4 − *p*2)/2.

For the *A* parameter, this index is used to represent an individual’s overall action/inaction preferences as a whole rather than generalized action/inaction preferences irrespective of norms and consequences. The mean action probability under the four situations could be calculated, i.e., *A* = (*p*1 + *p*2 + *p*3 + *p*4)/4. We do not think that the *I* parameter makes sense under sequential processing in the CNI model, and this is discussed below.

If the *C*/*N* parameter is greater (less) than 0, then the participants are identified as being sensitive to supporting (opposing) the *norm*/*consequence*. The larger the *C*/*N* parameter, the more sensitive it is to supporting the *norm*/*consequence*. If the *C*/*N* parameter is not significantly different to 0, then the participants are identified as not being sensitive to *norms*/*consequences*. The larger the *A* parameter, the more there is overall endorsement of the behavior proposal. If the *A* parameter is greater (less) than 0.5, then the participants are identified as having an overall action (inaction) preference. If the *A* parameter is not significantly different to 0.5 while at least one of the *C*/*N* parameters is significantly different to 0, the participants are identified as having a pure morality attitude that is utilitarian or deontological. If the *A* parameter is not significantly different to 0.5 while neither the *C* parameter nor the *N* parameter is significantly different to 0, the participants are identified as answering randomly.

To differentiate it from the CNI model, we named this new algorithm as “CAN.” To further demonstrate the reasonability of the CAN algorithm, we verified it in a more reasonable multinomial tree model. It showed that this model also supports our CAN algorithm.

### A More Reasonable Multinomial Process Tree Model Also Supports the CAN Algorithm

**FIGURE 2:**
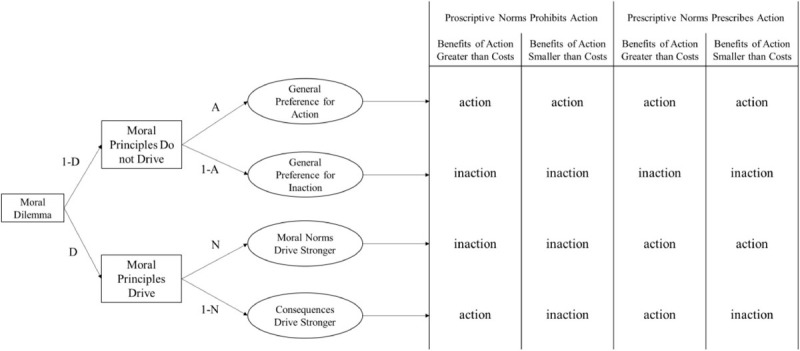
An alternative multinomial processing tree model constructed on the corrective dual-process model of morality. *D* denotes that the agent’s choices are driven by moral principles (norms or consequences), *N* denotes that the agent’s choices are driven more by norms. (1 – *N*) denotes that the agent’s choices are driven more by consequences. *A* denotes that the agent’s choices are driven by an overall preference for action.

Processing tree models are powerful frameworks to discuss potentially conflicted cognitive processes (Hutter and Klauer, 2016; Calanchini et al., 2018). Hence, an alternative MPT was constructed based on previous theoretical and empirical evidence (shown in Figure 2 and named “DNA model”). Bago and De Neys (2019) proposed a corrective dual-process model of moral cognition because they found that participants were intuitively utilitarian with a two-response paradigm. Furthermore, they concluded that the agent’s final moral judgment was dependent upon the absolute and relative strength between competing deontological and utilitarian intuitions (Bago and De Neys, 2019). Thus, in the MPT, we hypothesized that the driving forces from moral norms and consequences were parallel, and that the response pattern was dependent upon which driving force was stronger.

Together with the DNA model, four equations can be constructed:

(9) p1=(1-D)×A+D×(1-N)

(10) p2=(1-D)×A

(11) p3=(1-D)×A+D×N+D×(1-N)

(12) p4=(1-D)×A+D×N

In the DNA model, the consequence sensitivity and norm sensitivity can also be calculated algebraically. Consequence sensitivity can be represented by *D* × (1 − *N*) because the agent should be at first sensitive to moral principles, and then to the consequence principle. The algebraic expression of *D* × (1 − *N*) can be represented by (*p*1 − *p*2 + *p*3 − *p*4)/2 based on equations (9) to (12). Similarly, norm sensitivity can be represented by *D* × *N* and, further transformed from equations (9) to (12), it would be (*p*3 − *p*1 + *p*4 − *p*2)/2. These two indices were exactly identical to the CAN algorithm. Actually, the DNA model more approximately depicts the moral decision-making process according to the literature (Bago and De Neys, 2019; Neys and Pennycook, 2019).

As to the *I* parameter in the CNI model, it is problematic if we re-inspect it from the perspective of the DNA model. In the DNA model, the general preference for action irrespective of moral principles could be described as [(1 − *D*) × *A*], and its probability is depicted as *p*2 according to the equations. *p*2 portrays the probability when the agent endorses the behavioral proposal which is prohibited by both norm and consequence principles. Therefore, [(1 − *D*) × *A*] is more in line with the implication that the agent endorses the behavioral proposal irrespective of norms and consequences. Similarly, the general preference for inaction irrespective of moral principles could be described as [(1 − *D*) × (1 − *A*)], and its probability is depicted as (1 − *p*3). (1 − *p*3) portrays the probability when the agent declines the behavioral proposal which is advocated by both norm and consequence principles. Therefore, [(1 − *D*) × (1 − *A*)] is more in line with the implication that the agent declines the behavioral proposal irrespective of norms and consequences. Thus, in the DNA model, the generalized action preference irrespective of norms and consequences is *p*2, while the generalized inaction preference irrespective of norms and consequences is 1 − *p*3. As hypothesized by Gawronski et al. (2017), the sum of these two preference probabilities is 1. If that, *p*2 + (1 − *p*3) = 1, in turn, *p*2 = *p*3. It means that only when *p*2 = *p*3, the sum of the probabilities of generalized action and inaction preferences could be 1. However, it is obviously of little possibility. Hence, the fact that the *I* parameter in the CNI model claimed to depict the generalized inaction/action preferences irrespective of norms and consequences is not credible. On the contrary, the *A* parameter of the CAN algorithm could be easily understood and credible in methodological connotations as it depicts the *overall* action/inaction preferences among the four editions of moral dilemma.

Based on the above analyses, the CAN algorithm is theoretically tenable, and the *C/N/I* parameters, especially *N* and *I* parameters, obtained from the CNI model is systematically biased because of the sequential process presuppose. To further clarify the similarities and differences between the CAN algorithm and CNI model, we made direct mathematical contrasts as following.

### Mathematical Contrasts in Parameters Between the CNI Model and CAN Algorithm

The equations for the *C* parameter are identical under the two methods. With equations for the CNI model, we can transform them based on equations (1) and (2) into *C* = *p*1 − *p*2, and transform them based on equations (3) and (4) into *C* = *p*3 − *p*4. On average, *C* = (*p*1 − *p*2 + *p*3 − *p*4)/2. This equation is identical to the equation under the CAN algorithm. Therefore, we predict that in each study of Gawronski et al. (2017), the mean value of the *C* parameter under the CNI model will be almost equal to the mean values of *C* parameters under the CAN algorithm. Given that the *C* parameter under the CNI model was computed with maximum likelihood statistics at the group level whereas the C parameter under the CAN algorithm was computed with an algebraically subtracting strategy at the individual level, the mean values of these two parameters should be approximately equal rather than absolutely equal.

For the *N* parameter, we can transform equations (1) to (4) of the CNI model into *N* = (*p*3 − *p*1 + *p*4 − *p*2)/[2 × (1 − *C*)]. However, the equation for the *N* parameter in the CAN algorithm is *N* = (*p*3 − *p*1 + *p*4 − *p*2)/2. That is, the *N* parameter under the CAN algorithm divided by (1 − *C*) will be approximately equal to the *N* parameter under the CNI model. This is because the CNI model hypothesizes that the agent would consider the *norms* on the basis of not considering *consequences*. This precondition is untenable because it is entirely possible for the agent to consider *norms* first. Then, the sequential processing model should be the NCI model. If so, we can transform equations (5) to (8) of the NCI model into *N* = (*p*3 − *p*1 + *p*4 − *p*2)/2, which is identical to the CAN algorithm. Thus, we predict that *N*/(1 − *C*) under the CAN algorithm will be approximately equal to the *N* parameter value under the CNI model.

With regard to the *I* and *A* parameters, in the logic of the CNI model, the agent will consider the generalized action/inaction preference based on not considering *norms* and *consequences*. Similar to the *N* parameter, this precondition is not reasonable. The agent can have a generalized action/inaction preference to the behavior proposal first, and then her/his choices will be influenced by *norm* and *consequence* principles so that the choices are corrected based on the corresponding principles. Thus, the *I* parameter under the CNI model is not credible. We gave up the endeavor to identify the generalized inaction/action preferences irrespective of norms and consequences. Instead, the overall tendency of the agent’s action/inaction is more critical when reflecting the overall preferences. If the agent makes decisions purely according to *norm* and *consequence* principles, *p*2 would tend to be 0 and *p*3 would tend to be 1 because norm and consequence principles prohibit or advocate action; also the mean value of *p*1 and *p*4 would tend to be 0.5 because the *norm* principle and *consequence* principle are conflicted in terms of prohibiting or advocating action. Overall, the *A* parameter [i.e., (*p*1 + *p*2 + *p*3 + *p*4)/4] should have no differences to 0.5 if the agent makes decisions based purely on norm and consequence principles, or the agent is just answering randomly. Thus, the *A* parameter can represent the agent’s overall action/inaction preferences.

Given the similarities and differences of algebraic equations between the CNI model and CAN algorithm, we can draw some mathematical predictions which could be tested with the available data from the CNI model.

### Mathematical Predictions With the CNI Model and CAN Algorithm

Based on the methodological discussion above, we can make certain predictions. The *C* parameter under the CNI model will be approximately equal to the *C* parameter under the CAN algorithm (*H*1). The *N* parameter under the CNI model will be approximately equal to *N*/(1 − *C*) under the CAN algorithm (*H*2). The *I* parameter under the CNI model represents the agent’s generalized *inaction* versus *action* preferences, whereas the *A* parameter under the CAN algorithm represents the agent’s overall *action* versus *inaction* preferences. Thus, these two parameters will be reversed around 0.5. If the *I* parameter is higher than 0.5, the *A* parameter will be lower than 0.5, or *vice versa* (*H*3). Although the CNI model and CAN algorithm are algebraically different, the bias might be balanced systematically across between-subject conditions. Consequently, the tests of between-subject differences of the parameters generated from the CNI model and CAN algorithm could be almost identical (*H*4).

## Methods

In order to test the predictions, we re-analyzed the raw data of Gawronski et al. (2017) in which the CNI model was proposed and tested.

First, we downloaded the raw data of Gawronski et al. (2017) from https://osf.io/xt66w/. Then, we re-analyzed the raw data with the CNI model to ensure that the results of Gawronski et al. (2017) were reproducible. Our re-analysis results were identical to the results they reported.

Second, we used the CAN algorithm to generate *C*/*N*/*A* parameters, and also calculated *N*/(1 − *C*) with the mean values of *C* and *N* parameters. After that, we tested the hypotheses stated above.

Finally, because the *C*/*N*/*A* parameters generated from the CAN algorithm are at the individual level, these parameters could be used for correlation and other analyses. Thus, we tried to use Pearson’s correlation analysis between psychopathy scale rating and parameters in Study 4 of Gawronski et al. (2017).

## Results

With the CNI model, Gawronski et al. (2017) conducted four formal studies and one supplementary study. Each study was replicated based on recent concerns about the reproducibility of psychological findings (Open Science Collaboration, 2015). Thus, there were 10 studies in total. Study 1a/b discussed the gender differences in moral decision-making because it remained ambiguous in the traditional-dilemma approach (see Friesdorf et al., 2015). Study 2a/b explored the effects of cognitive load on moral decision-making (see Greene et al., 2008). Study 3a/b was on the effect of question-framing because one study demonstrated that personal force enhanced deontological responses (Greene et al., 2001). Study 4a/b explored the relationship between subclinical psychopathy level and utilitarian responses (Kahane et al., 2015; Bartels and Pizarro, 2011). Study S1a/b discussed the effects of harm salience on moral decision-making (see the relevant study, Conway and Gawronski, 2013).

**FIGURE A1:**
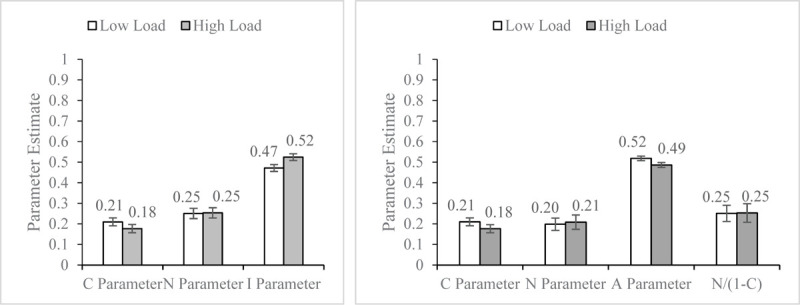
Results of Study 2a by Gawronski et al. (2017) obtained from the CNI model **(left)** and CAN algorithm **(right)**. Error bars represent ± 1 SE.

**FIGURE A2:**
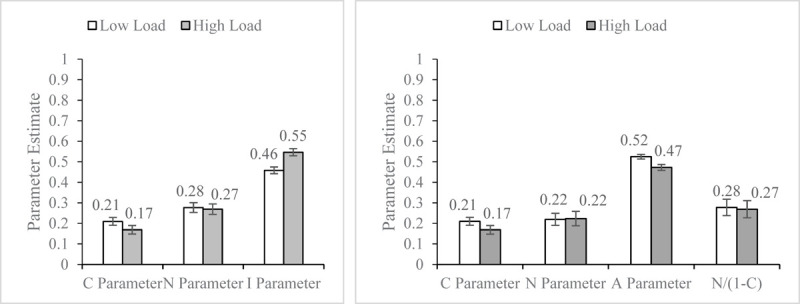
Results of Study 2b by Gawronski et al. (2017) obtained from the CNI model **(left)** and CAN algorithm **(right)**. Error bars represent ± 1 SE.

**FIGURE A3:**
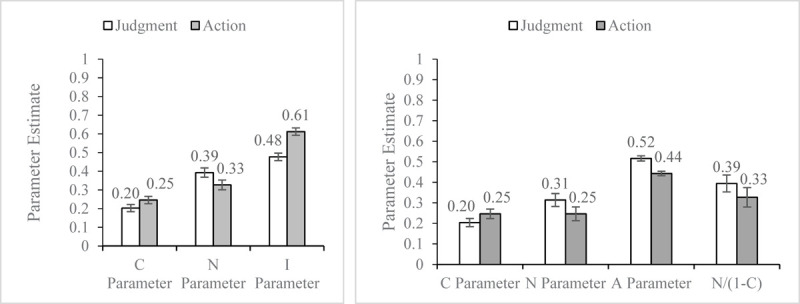
Results of Study 3a by Gawronski et al. (2017) obtained from the CNI model **(left)** and CAN algorithm **(right)**. Error bars represent ± 1 SE.

**FIGURE A4:**
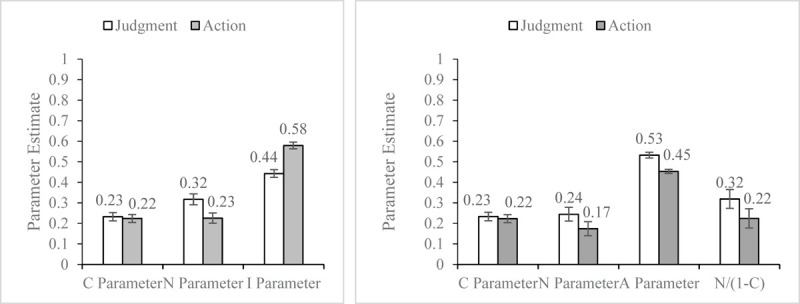
Results of Study 3b by Gawronski et al. (2017) obtained from the CNI model **(left)** and CAN algorithm **(right)**. Error bars represent ± 1 SE.

**FIGURE A5:**
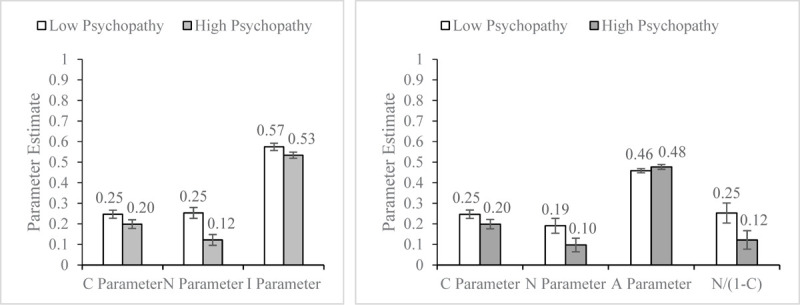
Results of Study 4a by Gawronski et al. (2017) obtained from the CNI model **(left)** and CAN algorithm **(right)**. Error bars represent ± 1 SE.

**FIGURE A6:**
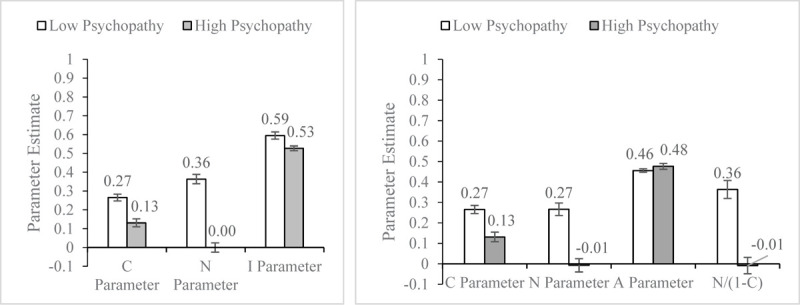
Results of Study 4b by Gawronski et al. (2017) obtained from the CNI model **(left)** and CAN algorithm **(right)**. Error bars represent ± 1 SE.

**FIGURE A7:**
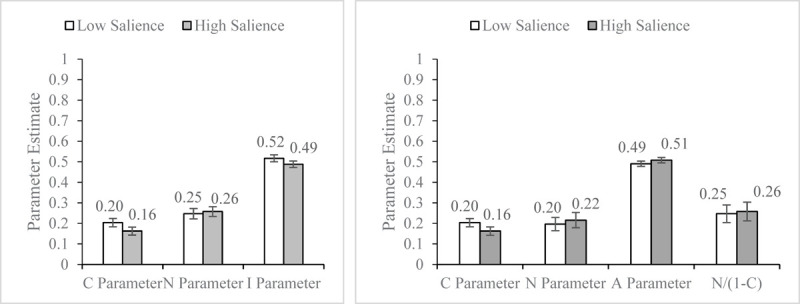
Results of Study S1a by Gawronski et al. (2017) obtained from the CNI model **(left)** and CAN algorithm **(right)**. Error bars represent ± 1 SE.

**FIGURE A8:**
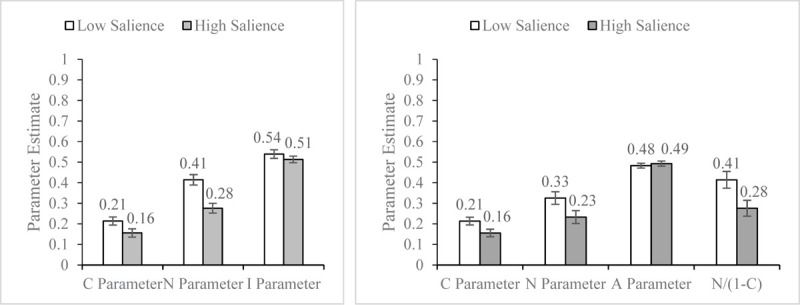
Results of Study S1b by Gawronski et al. (2017) obtained from the CNI model **(left)** and CAN algorithm **(right)**. Error bars represent ± 1 SE.

Gawronski et al. (2017) conducted analyses of a traditional dilemma, process dissociation (Conway and Gawronski, 2013), and the CNI model for each study. In our re-analysis, we replicated only the analysis of the CNI model and re-analyzed the raw data with the CAN algorithm. The patterns of the re-analysis results were almost identical across all studies. Hence, we present the results of Study 1a/1b; the remaining results are in the Appendices, Figures A1–A8 and Tables A1–A8.

**TABLE 1:** Test of gender differences with the CNI model and CAN algorithm in Study 1a by Gawronski et al. (2017).

	**Parameters**	**Results**	**Conclusion contrast**	**Results**	**Parameters**	
CNI model	*C*	Δ*G*^2^(1) = 1.34, *p* = 0.247, *d* = 0.164	Identical	*t*(199) = 1.08, *p* = 0.281, *d* = 0.153	*C*	CAN algorithm
	*N*	Δ*G*^2^(1) = 26.00, *p* < 0.001, *d* = 0.726	Identical	*t*(199) = 2.74, *p* = 0.007, *d* = 0.387	*N*	
	*I*	Δ*G*^2^(1) = 12.34, *p* < 0.001, *d* = 0.504	Identical	*t*(199) = 2.45, *p* = 0.015, *d* = 0.346	*A*	

### Test the Hypotheses of the Present Study

**FIGURE 3:**
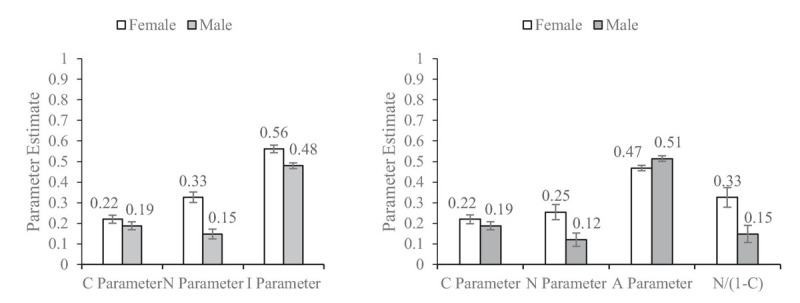
Results of Study 1a by Gawronski et al. (2017) obtained from the CNI model **(left)** and CAN algorithm **(right)**. Error bars represent ± 1 SE.

**FIGURE 4:**
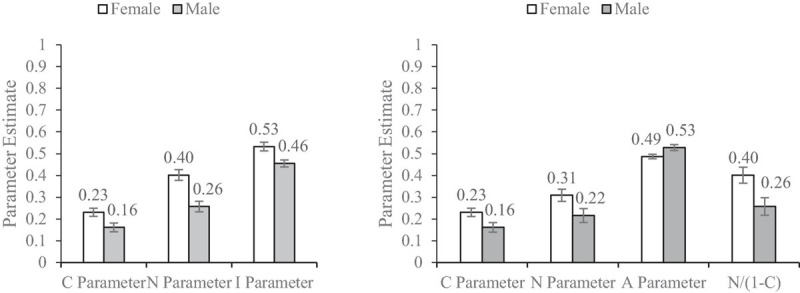
Results of Study 1b by Gawronski et al. (2017) obtained from the CNI model **(left)** and CAN algorithm **(right)**. Error bars represent ± 1 SE.

Across the 10 studies, all the predictions were validated, examples as shown in Figures 3, 4. The *C* parameter generated from the CNI model and CAN algorithm were approximately equal. The *N* parameter from the CAN algorithm was slightly smaller than that from the CNI model, and *N*/(1 − *C*) in the CAN algorithm was approximately equal to the *N* parameter in the CNI model. The *I* parameter under the CNI model and *A* parameter under the CAN algorithm would be reversed around 0.5 because their statistical implications were different. The larger the *I* parameter the more generalized the inaction *versus* action preferences, whereas the larger the *A* parameter the more overall action *versus* inaction tendencies there were. Furthermore, the differences in parameters across between-subject conditions were almost identical to the CNI model and CAN algorithm, as shown in Tables 1, 2. The independent sample *t*-test with the *C*/*A*/*N* parameters was more stringent than maximum likelihood statistics with the *C*/*N*/*I* parameters. Thus, a few marginally significant and low-significant results under the CNI model became non-significant under the CAN algorithm (see the Appendices).

**TABLE 2:** Test of gender differences with the CNI model and CAN algorithm in Study 1b by Gawronski et al. (2017).

	**Parameters**	**Results**	**Conclusion contrast**	**Results**	**Parameters**	
CNI model	*C*	Δ*G*^2^(1) = 6.43, *p* = 0.011, *d* = 0.364	Identical	*t*(195) = 2.39, *p* = 0.018, *d* = 0.153	*C*	CAN algorithm
	*N*	Δ*G*^2^(1) = 17.43, *p* < 0.001, *d* = 0.599	Identical	*t*(195) = 2.20, *p* = 0.029, *d* = 0.314	*N*	
	*I*	Δ*G*^2^(1) = 9.12, *p* = 0.003, *d* = 0.428	Identical	*t*(195) = 2.41, *p* = 0.017, *d* = 0.344	*A*	

### Demonstrating the Statistical Advantage of the CAN Algorithm

Moreover, the CAN algorithm could be used in correlation analysis. To demonstrate this statistical advantage over the CNI model, we ran a correlation analysis with the raw data of Study 4 a/b in Gawronski et al. (2017). They carried out the study in two sessions. In the first session, they recruited 522 (Study 4a) and 555 (Study 4b) participants to finish the psychopathy scale. Among the valid data of the first session, they identified 121 participants with psychopathy scores in the lowest quartile and 122 participants with psychopathy scores in the highest quartile (Study 4a). In the same way, they identified 138 participants with scores in the lowest quartile and 139 participants with scores in the highest quartile in Study 4b. In the second session (approximately 2 weeks after completion of the first session), the participants with psychopathy scores in the lowest quartile and highest quartile were invited to finish the moral dilemma part. The main purpose of Study 4a/b was to test whether participants with high versus low scores on the psychopathy measure differ in terms of *C/N/I* parameters. The results are shown in Tables A5, A6 in the appendices. Theoretically, if the *C/A/N* parameters significantly differ between the artificially divided low and high psychopathy conditions, the *C/A/N* parameters and the original continuous psychopathy scores might be significantly correlated.

We ran Pearson’s correlation analyses between their psychopathy scores and *C*/*A*/*N* parameters. In Study 4a, the psychopathy score was not correlated significantly with the *C* parameter (*r* = −0.122, *p* = 0.100), correlated significantly with the *N* parameter (*r* = −0.153, *p* = 0.038), and not correlated significantly with the *A* parameter (*r* = 0.106, *p* = 0.152). These results are congruent with tests on differences in *C*/*A*/*N* parameters, but incongruent with the test on differences in *C*/*I* parameters (see Table A5 in the appendices). In Study 4b, the psychopathy score was correlated significantly with the *C* parameter (*r* = −0.307, *p* < 0.001) and *N* parameter (*r* = −0.394, *p* < 0.001) but not correlated significantly with the *A* parameter (*r* = 0.103, *p* = 0.149). These results also supported the test results on differences based on the CAN algorithm, but did not support the test results on differences in the *I* parameter (Table A6 in the appendices).

Overall, the correlation analyses supported the results obtained from the differences test with *C*/*A*/*N* parameters while a little variated compared to the differences test with *C*/*N*/*I* parameters. Methodologically, the CAN algorithm generated the parameters at an individual level so that the parameters could be used for correlation and other common analyses, which is an advantage over the CNI model.

## Discussion

### Main Results of the Prior Predictions

All the hypotheses were supported. The *C* parameter under the CNI model and the *C* parameter under the CAN algorithm are approximately equal across all the studies, because the mathematical equations from the two methods are the same (*H1*). The *N* parameter under the CNI model is approximately equal to *N*/(1 − *C*) under the CAN algorithm in all the studies as predicted by the mathematical equation differences between the two methods (*H2*). As we can see that the value of (1 − *C*) is a number varying between 0 and 1, *N*/(1 − *C*) would be greater than the *N* value itself. That is, the *N* parameter generated from the CNI model is systematically overestimated in terms of the moral norm sensitivity. The value direction of the *I* parameter and *A* parameter is reversed around 0.5, as they ideally represent reversed implications. The results across all the studies supported this prediction (*H3*). However, we want to address again that the *I* parameter is biased. Its rationale is untenable which has been discussed in the introduction.

It is seemingly confusing that the statistical conclusions of the between-condition difference test are almost identical between the CNI model and CAN algorithm. Does this mean that these two methods are statistically equivalent? Absolutely not. We can easily tell that the mathematical equations are different between the *N* parameters and between the *I* and *A* parameters. These differences are among the within-subject condition, and would be systematically balanced in between-subject comparison. For example, in Figure 3, the *N* parameters for male and female under the CNI model are both overestimated in comparison with the *N* parameters under the CAN algorithm. Thus, when gender difference is tested, the overestimation biases are systematically balanced. Consequently, the tests of between-subject differences of the parameters generated from the CNI model and CAN algorithm become almost identical (*H4*). Nonetheless, the CAN algorithm and CNI model are fundamentally different.

Besides the hypotheses test, we ran another correlation analysis to demonstrate that the parameters obtained from the CAN algorithm could be used for correlation analyses which is an advantage over the CNI model. The *C*/*A*/*N* parameters are grounded at the individual level, so that they can be used for much more conventional analyses in a wide range of research designs.

### Conception Manipulation Development

The CAN algorithm is based on the CNI model in theoretical aspects. Originally, the traditional dilemma only considered a scenario in which proscriptive norms and benefits were greater than costs, such as the trolly-car paradigm (Foot, 1967) and footbridge paradigm (Thomson, 1976). Decades later, Conway and Gawronski (2013) explored two types of scenarios: consistent edition (proscriptive norms and benefits smaller than costs) and inconsistent edition (proscriptive norms and benefits greater than costs). Gawronski et al. (2017) varied the norms and consequences underlying the dilemma, and discussed four types of scenarios: a proscriptive/prescriptive norm with benefits greater/smaller than costs. The development of conception manipulation deepened insights on moral decision-making.

However, criticisms remain about the four-edition conception manipulation. Baron and Goodwin (2020) queried that, among the four editions of the same dilemma, the norms underlying the proscriptive editions and the prescriptive editions might not be the same moral norm. For example, in the dilemma of transplant, the norm of proscriptive editions is that *we should not harm other people*, whereas the norm of prescriptive editions is that *we should stop someone doing something harmful to others*. These two situations are essentially different on moral norms.

Nevertheless, we think the four-edition conception manipulation has contributed meaningfully. The conception manipulation is event-oriented but not moral principle-oriented. In the four editions of each dilemma, the event is consistent across editions but the underlying norms and consequences vary. Whether the agent is *sensitive* when the underlying norms and consequences change on the same event is important. Moreover, proscriptive and prescriptive morality are very important in people’s daily lives: they are the two facets of moral regulation (Janoff-Bulman et al., 2009). Thus, we agree that proscriptive scenarios and prescriptive scenarios have different norms, but we do not think it matters in the measurement of norm *sensitivity* in the conception manipulation aspect.

### The Methodological Development of the CAN Algorithm

The CAN algorithm reserved the theoretical conception manipulation development and also fixed the methodological limitations of the CNI model. The CNI model cannot be used for correlation analysis or multiple comparisons because the data obtained by the CNI model are represented at the group level rather than at the individual level. The CAN algorithm algebraically generates the parameters, and parameter data are represented at the individual level. Moreover, the CNI model is suitable only for binary categorical data, whereas the CAN algorithm can also be applied in continuous-scale data. Therefore, the *C*/*A*/*N* parameters can be used in a wide scope of research designs and data analyses.

The most serious methodologic limitation is that the CNI model presupposes that the agent sequentially considers consequences, norms, and generalized inaction/action preferences irrespective of norms and consequences. This precondition is questionable and leads to the *N* parameter being overestimated artificially. As the data re-analysis demonstrated, the *N* parameter under the CNI model approximately equaled *N*/(1 − *C*) under the CAN algorithm. The value of (1 − *C*) under the CAN algorithm was [0, 1], so the *N* parameter under the CNI model was systematically larger than the *N* parameter under the CAN algorithm. The sequential process presupposing of the CNI model also makes the *I* parameter dubious as it claimed to depict the extent of the agent’s generalized inaction/action preferences on the basis of not considering *consequences* or *norms*. Therefore, the CAN algorithm adopted a commonly used subtracting strategy to generate the *C* and *N* parameters and set up an overall action *versus* inaction preferences index: The *A* parameter.

Even though the agent processes moral decision-making sequentially, the NCI model is more credible than the CNI model. As demonstrated by the theoretical model of social intuition, people react emotionally first and revise their decisions cognitively later. People consider norms intuitively at first and consider consequences rationally later (Haidt, 2001). Therefore, the NCI model is more reasonable even if the style of the sequential process makes sense. However, increasing evidence implies that emotional and cognitive processes are parallel and independent rather than sequential (Greene, 2009; Cushman et al., 2010; Paxton and Greene, 2010; Hutcherson et al., 2015). Thus, the CAN algorithm is more appropriate for the demonstration of people’s moral preferences, especially *C* and *N* parameters.

### Possible Limitations of the CAN Algorithm

Although the CAN algorithm contributes to the literature by providing an individual level approach to compute the consequence, norm sensitivities, and overall action/inaction preferences in moral decision-making, potential limitations should be addressed in the future. The most significant limitation is that there is no statistical index at hand for potential measurement errors. It is difficult to discern whether the lack of correlations between trait measures and *C/A/N* parameters is due to measurement error versus genuine lack of a correlation. For the CAN algorithm, we are working to develop a statistically reasonable index to quantify the potential measurement errors.

Another limitation was also mentioned by Gawronski et al. (2017). There are only 24 moral dilemma trials, the parameter estimation might be deviated for the small number of observations. This limitation is the same for the original CNI model and CAN algorithm. More recently, they expanded 24–48 dilemma trials (Korner et al., 2020). We think this would be better to reduce the measurement deviation, although they did not fix the fundamental defect of the sequential processing presupposition.

## Conclusion

In summary, we addressed the methodological limitations of the CNI model and fixed these limitations with a new algorithm: the CAN. The CNI model presupposes that the agent sequentially considers consequences, norms, and generalized inaction/action preferences irrespective of consequences and norms in his/her moral decision-making process. We provided theoretical evidence that the decision-making process is more likely to be parallel with norms and consequences, and developed the CAN algorithm. The CNI model generated the parameters at the group level, and we calculated the parameters algebraically at the individual level so that the CAN algorithm was suitable for a larger range of research designs and conventional statistical analyses.

## Data Availability Statement

The original raw data of Gawronski et al. (2017) can be retrieved from https://osf.io/xt66w/. The syntax for re-analyses in present article can be retrieved from https://osf.io/65frg/. Further inquiries can bedirected to the corresponding author/s.

## Author Contributions

CL and JL developed the research idea together. CL analyzed the data and drafted the manuscript. JL supervised the research. Both authors revised the manuscript together and approved the submitted version.

## Conflict of Interest

The authors declare that the research was conducted in the absence of any commercial or financial relationships that could be construed as a potential conflict of interest.

## Footnotes

1 www.bertramgawronski.com/documents/CNI-Model_Materials.zip

## Appendices

Re-analyses of Study 2a by Gawronski et al. (2017).

**TABLE A1:** Test of differences in cognitive load with the CNI model and CAN algorithm in Study 2a by Gawronski et al. (2017).

	**Parameters**	**Results**	**Conclusion contrast**	**Results**	**Parameters**	
CNI model	*C*	Δ*G*^2^(1) = 1.35, *p* = 0.245, *d* = 0.168	Identical	*t*(192) = 1.22, *p* = 0.223, *d* = 0.175	*C*	CAN algorithm
	*N*	Δ*G*^2^(1) = 0.01, *p* = 0.927, *d* = 0.013	Identical	*t*(192) = 0.22, *p* = 0.827, *d* = 0.031	*N*	
	*I*	Δ*G*^2^(1) = 5.19, *p* = 0.023, *d* = 0.328	Identical	*t*(192) = 2.01, *p* = 0.045, *d* = 0.289	*A*	

Re-analyses of Study 2b by Gawronski et al. (2017).

**TABLE A2:** Test of differences in cognitive load with the CNI model and CAN algorithm in Study 2b by Gawronski et al. (2017).

	**Parameters**	**Results**	**Conclusion contrast**	**Results**	**Parameters**	
CNI model	*C*	Δ*G*^2^(1) = 2.08, *p* = 0.149, *d* = 0.209	Identical	*t*(192) = 1.45, *p* = 0.149, *d* = 0.209	*C*	CAN algorithm
	*N*	Δ*G*^2^(1) = 0.05, *p* = 0.826, *d* = 0.032	Identical	*t*(192) = 0.09, *p* = 0.927, *d* = 0.013	*N*	
	*I*	Δ*G*^2^(1) = 13.77, p < 0.001, *d* = 0.535	Identical	*t*(192) = 2.97, *p* = 0.003, *d* = 0.428	*A*	

Re-analyses of Study 3a by Gawronski et al. (2017).

**TABLE A3:** Tests of differences in question-framing with the CNI model and CAN algorithm in Study 3a by Gawronski et al. (2017).

	**Parameters**	**Results**	**Conclusion contrast**	**Results**	**Parameters**	
CNI model	*C*	Δ*G*^2^(1) = 2.44, *p* = 0.118, *d* = 0.230	Identical	*t*(184) = 1.39, *p* = 0.168, *d* = 0.203	*C*	CAN algorithm
	*N*	Δ*G*^2^(1) = 3.31, *p* = 0.069, *d* = 0.268	Basically identical	*t*(184) = 1.47, *p* = 0.144, *d* = 0.013	*N*	
	*I*	Δ*G*^2^(1) = 35.18, p < 0.001, *d* = 0.713	Identical	*t*(184) = 4.43, p < 0.001, *d* = 0.650	*A*	

Re-analyses of Study 3b by Gawronski et al. (2017).

**TABLE A4:** Test of differences in question-framing with the CNI model and CAN algorithm in Study 3b by Gawronski et al. (2017).

	**Parameters**	**Results**	**Conclusion contrast**	**Results**	**Parameters**	
CNI model	*C*	Δ*G*^2^(1) = 0.09, *p* = 0.767, *d* = 0.043	Identical	*t*(187) = 0.36, *p* = 0.721, *d* = 0.052	*C*	CAN algorithm
	*N*	Δ*G*^2^(1) = 6.15, *p* = 0.013, *d* = 0.363	Discrepant	*t*(187) = 1.46, *p* = 0.147, *d* = 0.212	*N*	
	*I*	Δ*G*^2^(1) = 29.50, p < 0.001, *d* = 0.799	Identical	*t*(187) = 4.62, p < 0.001, *d* = 0.673	*A*	

Re-analyses of Study 4a by Gawronski et al. (2017).

**TABLE A5:** Test of psychopathy differences with the CNI model and CAN algorithm in Study 4a by Gawronski et al. (2017).

	**Parameters**	**Results**	**Conclusion contrast**	**Results**	**Parameters**	
CNI model	*C*	Δ*G*^2^(1) = 2.77, *p* = 0.096, *d* = 0.247	Discrepant	*t*(182) = 1.56, *p* = 0.121, *d* = 0.230	*C*	CAN algorithm
	*N*	Δ*G*^2^(1) = 12.35, p < 0.001, *d* = 0.521	Basically identical	*t*(182) = 1.89, *p* = 0.060, *d* = 0.279	*N*	
	*I*	Δ*G*^2^(1) = 3.15, *p* = 0.076, *d* = 0.262	Discrepant	*t*(182) = 1.22, *p* = 0.223, *d* = 0.180	*A*	

Re-analyses of Study 4b by Gawronski et al. (2017).

**TABLE A6:** Test of psychopathy differences with the CNI model and CAN algorithm in Study 4b by Gawronski et al. (2017).

	**Parameters**	**Results**	**Conclusion contrast**	**Results**	**Parameters**	
CNI model	*C*	Δ*G*^2^(1) = 23.13, p < 0.001, *d* = 0.695	Identical	*t*(196) = 4.40, p < 0.001, *d* = 0.629	*C*	CAN algorithm
	*N*	Δ*G*^2^(1) = 111.80, p < 0.001, *d* = 1.48	Identical	*t*(196) = 6.12, p < 0.001, *d* = 0.875	*N*	
	*I*	Δ*G*^2^(1) = 8.90, *p* = 0.003, *d* = 0.406	Discrepant	*t*(196) = 1.28, *p* = 0.202^*a*^, *d* = 0.183	*A*	

*^*a*^Levene’s test was significant (p < 0.05), suggesting violation of the equal-variance assumption.*

Re-analyses of Study S1a by Gawronski et al. (2017).

**TABLE A7:** Test of differences in harm salience with the CNI model and CAN algorithm in Study S1a by Gawronski et al. (2017).

	**Parameters**	**Results**	**Conclusion contrast**	**Results**	**Parameters**	
CNI model	*C*	Δ*G*^2^(1) = 2.15, *p* = 0.143, *d* = 0.211	Identical	*t*(193) = 1.42, *p* = 0.156, *d* = 0.204	*C*	CAN algorithm
	*N*	Δ*G*^2^(1) = 0.10, *p* = 0.758, *d* = 0.044	Identical	*t*(193) = 0.39, *p* = 0.696, *d* = 0.056	*N*	
	*I*	Δ*G*^2^(1) = 1.60, *p* = 0.206, *d* = 0.182	Identical	*t*(193) = 0.97, *p* = 0.334, *d* = 0.139	*A*	

Re-analyses of Study S1b by Gawronski et al. (2017).

**TABLE A8:** Test of differences in harm salience with the CNI model and CAN algorithm in Study S1b by Gawronski et al. (2017).

	**Parameters**	**Results**	**Conclusion contrast**	**Results**	**Parameters**	
CNI model	*C*	Δ*G*^2^(1) = 4.40, *p* = 0.036, *d* = 0.305	Identical	*t*(189) = 2.20, *p* = 0.029, *d* = 0.204	*C*	CAN algorithm
	*N*	Δ*G*^2^(1) = 15.79, p < 0.001, *d* = 0.580	Identical	*t*(189) = 2.11, *p* = 0.036, *d* = 0.305	*N*	
	*I*	Δ*G*^2^(1) = 0.97, *p* = 0.325, *d* = 0.144	Identical	*t*(189) = 0.58, *p* = 0.563, *d* = 0.084	*A*	
